# Alcohol increases inattentional blindness when cognitive resources are not consumed by ongoing task demands

**DOI:** 10.1007/s00213-017-4772-9

**Published:** 2017-11-02

**Authors:** Alistair J. Harvey, Sarah J. Bayless, Georgia Hyams

**Affiliations:** 1 Department of Psychology, University of Portsmouth, King Henry I Street, Portsmouth, PO1 2DY UK; 2 Department of Psychology, University of Winchester, Winchester, SO22 4NR UK

**Keywords:** Alcohol intoxication, Visual attention, Inattentional blindness, Task performance

## Abstract

**Rationale:**

Inattentional blindness (IB) is the inability to detect a salient yet unexpected task irrelevant stimulus in one’s visual field when attention is engaged in an ongoing primary task. The present study is the first to examine the impact of both task difficulty and alcohol consumption on IB and primary task performance.

**Objectives:**

On the basis of alcohol myopia theory, the combined effects of increased task difficulty and alcohol intoxication were predicted to impair task performance and restrict the focus of attention on to task-relevant stimuli. We therefore expected increases in breath alcohol concentration to be associated with poorer primary task performance and higher rates of IB, with these relationships being stronger under hard than easy task conditions.

**Methods:**

This hypothesis was tested in a field study where alcohol drinkers in a local bar were randomly assigned to perform a dynamic IB task with an easy or hard visual tracking and counting task at its core (Simons and Chabris in Perception 28:1059–1074, 1999).

**Results:**

Increasing the difficulty of the primary task reduced task accuracy but, surprisingly, had no impact on the rate of IB. Higher levels of alcohol intoxication were, however, associated with poorer task performance and an increased rate of IB, but only under easy primary task conditions.

**Conclusions:**

Results are consistent with alcohol myopia theory. Alcohol intoxication depletes attentional resources, thus reducing the drinker’s awareness of salient stimuli that are irrelevant to some ongoing primary task. We conclude that this effect was not observed for our hard task because it is more resource intensive, so leaves no spare attentional capacity for alcohol to deplete.

There is a good deal of evidence supporting *alcohol myopia theory*, the idea that alcohol consumption depletes attentional resources inducing a form of “short-sightedness” through which only the most central or important environmental cues are processed (Steele and Josephs 1990). In the perception and cognition literature, for example, small to moderate doses of alcohol have been found to slow visual search times for peripheral targets (Hoyer et al. 2007; Moskowitz and Sharma 1974), restrict visual scanning to central and semantically salient image features (Harvey 2014; Harvey et al. 2013a; Moser et al. 1998) and impair attention and memory for peripheral stimuli (Bayless and Harvey 2017; Canto-Pereira et al. 2007; Harvey 2016; Schreiber Compo et al. 2011; Schulte et al. 2001). Yet, the theory offers no predictions concerning the attentional focus of drinkers faced with competing salient stimuli.

In the case of face recognition and studies of eyewitness identification, for instance, alcohol is rarely shown to impair performance which, given the salience of the target faces in these tasks, is consistent with AMT (Hagsand et al. 2013; Harvey 2014; Harvey et al. 2013b; Kneller and Harvey 2016). But if an assailant were to suddenly draw a gun—on to which stimulus is the attention of a drunken witness now expected to narrow? AMT is similarly challenged by salient events that emerge when the drinker’s attention is engaged elsewhere. Take the example of an intoxicated driver struggling to negotiate a complex road system when a friend waves from the kerbside ahead. Is the driver’s attention immediately captured by the unexpected gesture or does her ‘myopic’ focus on vehicle control reduce her odds of spotting it?

A question of this form was addressed by Clifasefi et al. (2006) who studied the impact of alcohol intoxication on inattentional blindness (IB)—the common human failure to perceive a novel but unexpected visual object in plain view when attention is otherwise engaged. Using an IB paradigm developed by Simons and Chabris (1999), Clifasefi and colleagues had a group of sober and intoxicated participants view video footage of two basketball teams each passing a ball among fellow team members. The primary experimental task is to keep a running count of the passes made by one team; however, a few seconds into the game, an unexpected female in a gorilla suit casually strolls among the players and across the court. When the video clip ends, participants report their total pass count then the experimenter asks if they noticed anything unusual about the clip. In the original study 44% of participants spotted the gorilla, reflecting a surprisingly high incidence of IB to a salient visual stimulus (Simons and Chabris 1999). In their intoxicated group, however, Clifasefi et al. observed a gorilla spotting rate of only 18%, compared to the more typical rate of 46% shown by the sober group.

This finding suggests that alcohol narrows the viewer’s focus on to one functionally salient aspect of a visual scene (i.e. tracking the number of white team passes) while leaving insufficient resources to monitor its wider aspects, which increases the likelihood that some visually salient yet task-irrelevant feature will be missed. But the extent to which Clifasefi et al.’s (2006) participants were centred on the primary task is unclear as no measure of pass-counting accuracy was reported. It would also be useful to know whether raising the attentional demands of the primary task further increases the rate of IB in intoxicated participants by intensifying alcohol myopia.

One way to test this resource depletion account of AMT and IB is to vary the *perceptual load* imposed by the primary task. According to Cartwright-Finch and Lavie (2007), perceptual load can be increased either by adding more display features that are relevant to the target stimulus (e.g. such as increasing the number of target-similar distractor items in a visual search task), or by manipulating task demands across identical visual displays (e.g. have participants identify either the presence/absence [low load] or the size/position [high load] of a target within the same array of distractors). Primary tasks with a high perceptual load have been shown to increase IB rates relative to tasks with a low perceptual load (Cartwright-Finch and Lavie 2007; Simons and Chabris 1999; Simons and Jensen 2009) and these findings are explained by Lavie’s (1995) *perceptual load theory*. According to this view, a limited capacity attentional system strictly prioritises the processing of task-relevant stimuli and allocates resources to task irrelevant processing *only* when there is capacity to spare. When engaged in a primary task with a high perceptual load, viewers are therefore unlikely to have sufficient cognitive resources to detect even the most salient but unexpected task-irrelevant stimuli.

In the present study, we therefore extend the work of Clifasefi et al. (2006) by adding a measure of primary task accuracy and a task difficulty manipulation, to see if an increase in perceptual load produces higher rates of IB. In addition to the easy version of the Simons and Chabris (1999) task, where viewers maintain a single tally of white team passes, we assigned half our participants to a more challenging version requiring them to keep separate counts of the number of aerial and bounce passes made by the same team. The impositions of discriminating, tracking and tallying two different types of ball pass increase the task’s perceptual load and thus the attentional burden placed on participants.

On this basis, with the effects of alcohol intoxication on primary task performance statistically controlled, we predicted poorer primary task performance and less noticing of unexpected stimuli among participants who performed the hard task, compared to those assigned the easy task. More importantly, in line with AMT, we hypothesised that increases in breath alcohol concentration would be significantly predictive of both poorer primary task performance and higher rates of IB, with these negative associations expected to be larger under hard task than easy tasks conditions.

## Method

### Participants

A convenience sample of 104 patrons of the host university’s Student Union bar volunteered to take part in this field experiment (50 males and 54 females) aged 18–30 years (*M* = 20.46, SD = 1.96). All reported normal or corrected-to-normal vision.

This sampling was on the basis of two a priori power analyses using G*Power (Faul et al. 2009). The first of these indicated that a total sample size of at least 67 participants was required for an 80% chance of detecting a moderate sized task difficulty effect of *f* = .35, using an ANCOVA test with one covariate (BAC, in this case), with an alpha of .05. For detecting the relationship between BAC and task performance, and between BAC and IB, the second G*Power analysis revealed that 49 participants per task group were required for 80% power to detect correlations of moderate strength (*r* = .35), again with an alpha of .05.

### Design

A one-way between-subjects design was used to test the effect of task difficulty on primary task performance and IB. Task difficulty served as the independent variable with each participant being randomly assigned to either the easy or hard version of the Simons and Chabris (1999) task. Primary task performance was a percentage measure of pass-counting accuracy and IB was measured by a count of the number of unexpected stimuli each participant missed while performing the pass-counting task. Breath/blood alcohol concentration was treated as a covariate within this experimental design. Simple regression models were also employed to explore the extent to which variations in breath/blood alcohol concentration predict primary task performance and IB under easy and hard task conditions.

### Materials and apparatus

Breath alcohol was measured using an Alcosense DA5000 Pro Digital Breathalyser with the unit reported by the device (mg/100 ml) converted to an estimate of blood alcohol concentration (BAC, % by volume) using a 2100:1 blood/breath ratio. In an attempt to produce a more robust measure of IB than merely the failure to spot one unexpected stimulus we used Simons’ (2010) *Monkey Business Illusion*, a modified version of the Simons and Chabris (1999) clip in which two additional unexpected stimulus events coincide with the gorilla’s appearance. The clip features two female teams (one wearing black shirts and the other, white) each passing and bouncing one basketball to fellow team members on a small stage. The two balls are kept in play throughout the game, which lasts 30 s. A gorilla-suited female enters stage right at 17 s, a member of the black shirt team exits stage right at 18 s, and the curtain drawn behind the stage gradually changes in colour from red to gold during the 17- to 20-s period.

### Procedure

The study was administered with full adherence to the British Psychological Society Code of Ethics and Conduct and was approved by the host university’s ethics committee. Drinkers in the host university’s Student Union bar were approached between 6 and 9 pm and asked if they wished to participate in a study for the Department of Psychology. All those who volunteered could communicate clearly and showed no signs of extreme intoxication such as slurred speech, anger, boisterousness, confusion, nausea or stupor. Each volunteer was taken individually to a quiet meeting room one floor above the Union bar to complete the experiment, which was always pre-booked to ensure exclusive access for data collection. Each participant was seated at the table and given the study information sheet and consent form. After signing to consent, they were breathalysed then asked to provide a subjective rating of their intoxication level on a scale ranging from 1 (completely sober) to 10 (extremely intoxicated). Following these alcohol measures, the experimenter placed a 15″ laptop computer in front of the participant on which the Simons (2010) clip described above was cued to play. Prior to viewing the clip, each participant was instructed to count, as accurately as possible, either the total number of passes (easy task) or the number of aerial and bounce passes (hard task) made by the white-shirt team, depending on which version of the task they had been assigned. Participants completing the “easy task” were told that a pass occurs when one team member passes the ball to a fellow team member. Participants completing the “hard task” were told that an aerial pass is when a player throws the ball to a fellow team member without the ball touching the ground, and that a bounce pass is when the ball bounces on the ground as it is passed from one team player to another. After viewing the clip, participants were first asked to report their pass count(s) and were then asked if they had noticed anything unusual about the game. In cases where unexpected stimuli were spotted, the experimenter made a note of these (gorilla, curtain change, player exiting or some combination thereof). After reporting on the stimulus scene, participants were given a second breath test and asked if they had encountered the gorilla video before. Four confessed to having prior knowledge of the clip so data from these participants were discarded. Finally, participants were debriefed and thanked for volunteering. In order to preserve the naivety of prospective participants, they were asked not to discuss the study with fellow drinkers upon their return to the bar.

## Results

### Intoxication levels

**Fig. 1:**
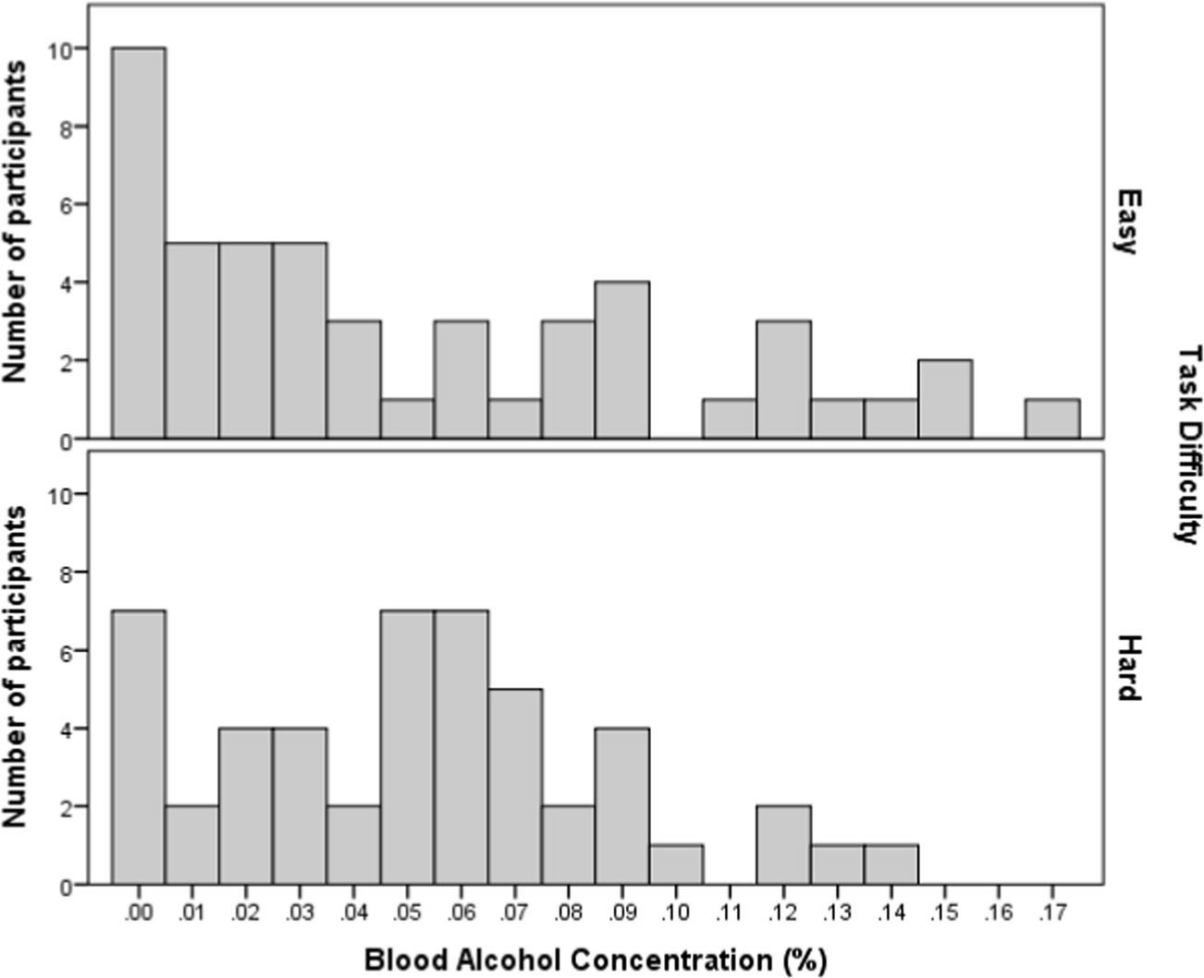
Distribution of blood alcohol concentration for the easy and hard task group

**Fig. 2:**
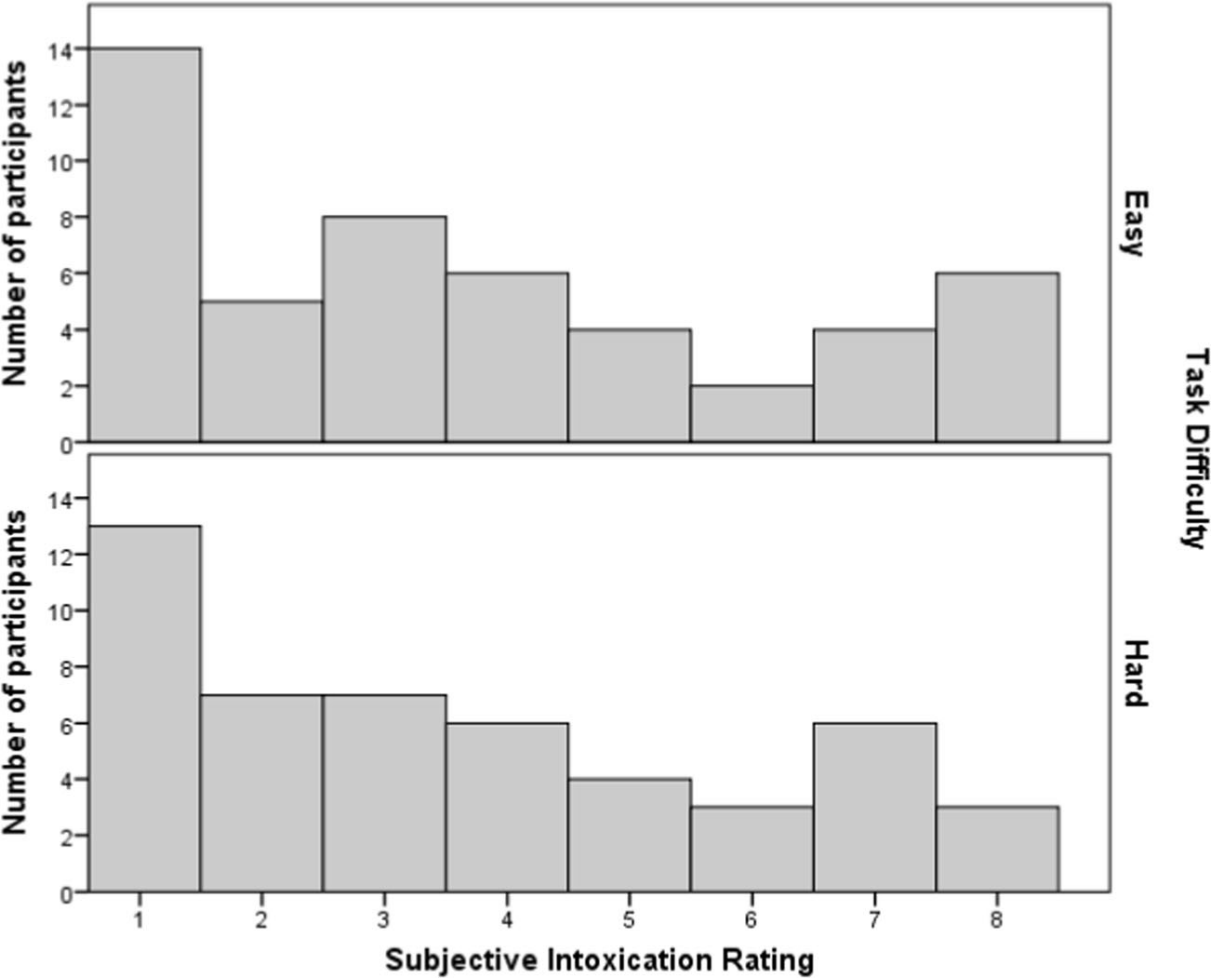
Distribution of subjective intoxication ratings for the easy and hard task group

Figure 1 shows the range of mean BACs (averaged across the two breath measures) for easy and hard task participants. The distribution for the easy group is positively skewed with a mode of 0.00% (*n* = 10) and a mean of 0.05% (SD = 0.05). The distribution for the hard task group is tri-modal (0.00, 0.05 and 0.06%) but shares the same mean (*M* = 0.05%; SD = 0.04) and is also positively skewed. Distributions for the subjective intoxication ratings are shown in Fig. 2, again as a function of task difficulty. These have a strong positive skew, a prominent mode of 1 and comparable means for the easy (*M* = 3.67, SD = 2.47) and hard task (3.59, SD = 2.34).

### Primary task accuracy

Our measure of easy task accuracy is the participant’s total pass count as a proportion of the actual number of white team passes (16). For the hard task, it is the sum of the participant’s bounce-pass count as a proportion of the actual bounce-pass count (6) and aerial-pass count as a proportion of the actual aerial-pass count (10). These scores are expressed as percentages with a percentage deduction calculated for counts *over* the actual number of passes made. For example, an easy task pass count of 18 (2 over the actual number of passes) produced a percentage deduction of 12.5% (2/16*100), yielding an accuracy score of 87.5% (100–12.5).

Overall mean pass-counting accuracy was 85.27% (SD = 21.84) reflecting high levels of engagement with the primary task. Two cases (one from each task group) were excluded from further analysis due to accuracy scores more than 3 SDs below the mean. As expected, remaining participants in the easy task group showed a significantly higher mean pass-counting accuracy (91.98%, SD = 16.73) than those in the hard task group (82.68%, SD = 15.06), *F*(1, 96) = 8.367, *p* = .005. However, the majority of participants had been drinking alcohol prior to the experiment, therefore a one-way analysis of covariance was used to examine the effect of task difficulty on primary task performance while controlling for the effect of intoxication on pass-counting performance. Even with task accuracy scores adjusted to account for the influence of alcohol intoxication, the difference in accuracy between the easy (*M* = 91.97, 95% CI [86.59, 96.31]) and hard group (*M* = 82.68, 95% CI [78.30, 87.06]) remained highly significant, *F* (1, 95) = 8.864, *p* = .004, *n*_p_^2^ = .09.

In order to explore the extent to which alcohol (BAC) predicts performance on the easy and hard tasks, a simple regression was conducted on each group’s data, with BAC serving as the predictor variable and pass-counting accuracy as the outcome variable. For the easy task, as expected, alcohol intoxication was a highly significant predictor of primary task performance, with 18% of the score variance accounted for by changes in BAC (*R* ^2^ = .179), *β* = − .423, *t*(47) = − 3.204, *p* = .002. Surprisingly, though, alcohol intoxication was not a significant predictor of pass-counting performance among the hard task group, *β* = − .018, *t*(47) = − .122, *p* = .903. Despite this contrasting association of BAC with easy and hard task performance, it should be noted that the interaction between our covariate (BAC) and task group was not significant in the ANCOVA model presented above, *F*(1, 94) = 3.262, *p* = .074, thus confirming sufficiently homogenous regression slopes for this analysis.

### Inattentional blindness

Of the 98 participants included for analysis, 43 (43.88%) spotted the gorilla, 2 (2.04%) spotted the curtain change colour and 6 (6.12%) spotted the departing player. Of these spotters, only 6 noticed more than one unexpected event (5 spotted the gorilla and the exiting player; 1 spotted the gorilla and the curtain change). IB was scored on a scale of 0–3, reflecting the number of unexpected stimulus events missed. The incidence of IB was only slightly higher for the hard task group (*M* = 2.55, SD = .61, 95% CI [2.38, 2.71]) than the easy task group (*M* = 2.41, SD = .61, 95% CI [2.23, 2.57]) and, contrary to expectation, a one-way analysis of variance revealed this difference to be non-significant, *F*(1, 96) = 1.335, *p* = .251. Furthermore, this null effect of task difficulty on IB remained unchanged in an ANCOVA model where IB scores were adjusted to control for the effect of intoxication (BAC), *F*(1, 95) = 1.405, *p* = .239, *n*_p_^2^ = .02.

As for our primary task analysis, we ran simple regressions on easy and hard group data, only this time to examine the extent to which alcohol intoxication (BAC) predicts IB at each level of task difficulty. As hypothesised, alcohol intoxication was found to be a highly significant predictor of IB in the easy condition, accounting for 13% of the IB variance for this task group (*R* ^2^ = .13), *β* = .361, *t*(47) = 2.650, *p* = .011. But, contrary to prediction, BAC was revealed to be a weak and non-significant predictor of IB in the hard task condition, accounting for less than 2% of the IB score variance (*R* ^2^ = .014), *β* = .117, *t*(47) = .809, *p* = .423. However, the interaction between the covariate (BAC) and task group in the IB ANCOVA was non-significant, *F*(1, 94) = .704, *p* = .403, suggesting that the homogeneity of regression slopes assumption for the second ANCOVA model was not violated.

We should add that the pattern of inferential findings reported above hold when we substitute BAC with our subjective measure of alcohol intoxication.

## Discussion

This study is the first to examine the relationship between alcohol intoxication and task difficulty on primary task performance and rates of IB in a dynamic, real-world IB paradigm (Simons and Chabris 1999). The key findings are summarised as follows. Increasing task difficulty had a significant adverse effect on task performance that was independent of the influence of alcohol intoxication. Contrary to our hypothesis, however, the task difficulty manipulation had no effect on our measure of IB. Moreover, although alcohol intoxication was associated with a decrease in primary task accuracy and an increase in IB, these expected effects were shown only for the easy version of our primary task. This finding nevertheless aligns with the work of Clifasefi et al. (2006), who observed a similar effect of alcohol intoxication on gorilla spotting using the same “easy” Simons and Chabris’ (1999) ball-pass counting task, thus lending further support to the attention-narrowing account of AMT. According to this view, alcohol depletes attentional resources with those leftover being prioritised for processing only the most important, task-relevant features of the visual field. This channelling of attention effectively narrows the scope of peripheral monitoring making it harder for the intoxicated viewer to detect an unexpected task irrelevant stimulus—even one as salient as a fake gorilla amidst a basketball game.

Why alcohol did not impair counting performance or increase IB among our hard task group is less obvious. We suggest that the mean level of intoxication in our sample—relatively low for a field study of this nature (BAC ≈ .05%) and possibly associated with our early evening test slots—was not high enough to significantly impair primary task performance. Yet, it is quite possible that the additional demands of maintaining two separate pass counts left hard task participants with no spare attentional resources for alcohol to deplete. If so, we should expect intoxication to have a smaller impact on IB, and peripheral attention generally, in studies where the attentional demands of the primary task are high.

We have recently reported an example of such a study in Bayless and Harvey (2017), where an increase in difficulty of a dual (central/peripheral) attentional task impaired the performance of a sober group but not their intoxicated counterparts. Participants of this study were told to fix their gaze on a centrally positioned fixation cross and count its flashes as an array of coloured circles was displayed around this focal point in the screen’s periphery. On each trial, one of the peripheral circles was cued by a small arrow, also positioned in the screen’s centre and, following a blank screen interval, participants had to decide if the single circle presented next was either the same or different in colour to the one just previously cued. For trials showing four circles in the peripheral array, sober performance was superior to the alcohol group, and particularly so for peripheral recognition, which is obviously consistent with AMT. However, the sober group’s peripheral recognition advantage was significantly reduced for six-circle trials while the alcohol group’s performance was unaffected by the increase in array size. Although the Bayless and Harvey (2017) task is somewhat different to that of the present study, this earlier result of ours is at least consistent with the idea that alcohol will probably not worsen peripheral awareness when the drinker’s cognitive resources are already fully deployed with the demands of an ongoing task.

Another surprising feature of the present study is the absence of a significant effect of task difficulty on IB, which is inconsistent with previous studies (e.g. Cartwright-Finch and Lavie 2007; Lavie 1995; Simons and Chabris 1999; Simons and Jensen 2009). Our only explanation for this is that the lower accuracy scores of the hard group reflect a waning focus on this more challenging primary task, which produced attentional drifts away from it and thus a noticing rate for task irrelevant events comparable to that of the easy group. This view is supported by the fact that attentional drift or *mind wandering*, while more commonly experienced during mundane tasks, has been shown to cause greater impairment to the performance of difficult tasks (Feng et al. 2013). Mind wandering is also known to be associated with low attentional (or working memory) capacity (Kane et al. 2007) and, interestingly, is more likely to occur when participants are alcohol intoxicated than when sober (Sayette et al. 2009). We nevertheless acknowledge that this attentional drift account is speculative and we are currently examining the effects of alcohol on IB in a laboratory-based study with eye-tracking and a more tightly controlled computer-based IB task to shed further light on this issue.

So what does the present study teach us about the attentional focus of the drunken driver or the intoxicated witness? On the basis of our easy task data, we draw the same conclusion as Clifasefi et al. (2006), namely, that intoxicated viewers cognitively engaged with one aspect of their visual environment—such as traversing a chaotic road junction or anticipating the actions of an armed assailant—are less likely than sober counterparts to notice other unexpected scene developments, even if these events are novel and occur in plain sight. But our hard task data suggest that the extent to which alcohol increases IB is also determined by the attentional demands of the primary task. Should this be so burdensome as to consume all of the viewer’s mental resources—such as driving on ice perhaps, or convincing an armed assailant not to shoot—then we expect any additional attentional narrowing effects of intoxication to be negligible, as alcohol cannot drain resources from an empty cognitive reserve.

Unfortunately, though, as our participants were required to focus on the primary task continuously and *prior* to presentation of the unexpected stimulus, the present study reveals little about the attentional orientation of drinkers suddenly and simultaneously presented with objects equally matched in importance or goal relevance. Anticipating which of multiple external stimuli drinkers will prioritise remains a considerable challenge, not least because a fluid range of internal cues are also likely to be competing for their awareness. Previous basic research on perception and memory has emphasised the importance of arousal as a mechanism for biasing the viewer’s attention, facilitating swift selection of one of a number of compelling visual stimuli (see Mather and Sutherland 2011, for a review). But the matter is further complicated by the possibility that our viewer may attend not to one specific spatial location but to a stimulus distributed across the visual field, such as an object superimposed by other less important items, or perhaps to multiple objects grouped on the basis of some gestalt principle (Duncan 1984; Emmanouil and Magen 2014; O’Craven et al. 1999). Visual attention and memory researchers should therefore consider carefully the extent to which critical stimuli elicit participant arousal in future alcohol studies and ensure that these target objects are not always confounded with their spatial location.

On the basis of the present findings, we conclude that visual awareness of a salient but task-irrelevant stimulus is significantly less likely following alcohol intoxication if the drinker is engaged in an ongoing primary task, but only when his attentional capacity is *not* already exhausted by that task’s perceptual and cognitive demands.
